# Multiple Myeloma Patient Tumors With High Levels of Cereblon Exon-10 Deletion Splice Variant Upregulate Clinically Targetable Pro-Inflammatory Cytokine Pathways

**DOI:** 10.3389/fgene.2022.831779

**Published:** 2022-02-09

**Authors:** Kubra Karagoz, Matthew Stokes, María Ortiz-Estévez, Fadi Towfic, Erin Flynt, Sarah Gooding, William Pierceall, Anjan Thakurta

**Affiliations:** ^1^ Translational Medicine, Bristol Myers Squibb, Summit, NJ, United States; ^2^ Bristol Myers Squibb Center for Innovation and Translational Research Europe, Sevilla, Spain; ^3^ Bristol Myers Squibb, San Diego, CA, United States; ^4^ MRC Molecular Haematology Unit, Weatherall Institute of Molecular Medicine, University of Oxford, Oxford, United Kingdom; ^5^ Department of Haematology, Oxford University Hospitals NHS Trust, Oxford, United Kingdom; ^6^ NIHR Oxford Biomedical Research Centre, University of Oxford, Oxford, United Kingdom; ^7^ Oxford Centre for Translational Myeloma Research, University of Oxford, Oxford, United Kingdom

**Keywords:** multiple myeloma, immunomodulatory drugs, drug resistance, cereblon (CRBN), exon-10, venetoclax, tazemetostat

## Abstract

Immunomodulatory drugs (IMiDs), including lenalidomide and pomalidomide, are used in the routine treatment for multiple myeloma (MM) patients. Cereblon (CRBN) is the direct molecular target of IMiDs. While CRBN is not an essential gene for MM cell proliferation, the frequency of CRBN genetic aberrations, including mutation, copy number loss, and exon-10 (which includes a portion of the IMiD-binding domain) splicing, have been reported to incrementally increase in later-line patients. CRBN exon-10 splicing has also been shown to be associated with decreased progression-free survival in both newly diagnosed and relapsed refractory MM patients. Although we did not find significant general splicing defects among patients with CRBN exon-10 splice variant when compared to those expressing the full-length transcript, we identified upregulated TNFA signaling via NFKB, inflammatory response, and IL-10 signaling pathways in patients with exon-10 splice variant across various data sets—all potentially promoting tumor growth via chronic growth signals. We examined master regulators that mediate transcriptional programs in CRBN exon-10 splice variant patients and identified BATF, EZH2, and IKZF1 as the key candidates across the four data sets. Upregulated downstream targets of BATF, EZH2, and IKZF1 are components of TNFA signaling via NFKB, IL2/STAT5 signaling pathways, and IFNG response pathways. Previously, BATF-mediated transcriptional regulation was associated with venetoclax sensitivity in MM. Interestingly, we found that an EZH2 sensitivity gene expression signature also correlated with high BATF or venetoclax sensitivity scores in these tumors. Together, these data provide a rationale for investigating EZH2 inhibitors or venetoclax in combination with the next generation CRBN-targeting agents, such as CELMoDs, for patients overexpressing the CRBN exon-10 splice variant.

## Introduction

Although the target of immunomodulatory drugs (IMiDs)—cereblon (CRBN)—is not an essential gene in multiple myeloma (MM) cells, genomic defects in CRBN, including mutation, copy number loss, and a specific exon-10–deleted splice transcript variant (henceforth called *CRBN-Del-Exon10*), increase in IMiD-resistant relapsed and refractory MM (RRMM) patients (Ito et al., 2010; Lopez-Girona et al., 2012; Thakurta et al., 2014; Kortum et al., 2016; Franssen et al., 2018; Gooding et al., 2021). The CRBN gene contains 11 exons encoding a protein comprising 442 amino acid residues with its C-terminal portion (encoded in part by exon-10) containing the drug-binding domain. *CRBN-Del-Exon10* variant was previously associated with lenalidomide resistance (Chamberlain et al., 2014; Gandhi et al., 2014). Unlike mutation or copy loss, *CRBN-Del-Exon10* splice variant was observed in newly diagnosed MM (NDMM) patients at ∼2.9% prevalence which increased to 29.6% in IMiD-resistant RRMM and was a prognostic biomarker for poor outcome in both disease settings (Gooding et al., 2021). However, the biological basis for its prognostic and/or predictive role (e.g., as a biomarker of resistance to IMiDs) is still not known. There are multiple factors that may contribute to these observed clinical effects. The potential deletion of the drug-binding region may lead to a direct loss of drug-binding function of CRBN; however, it is unclear if the relative amount of full-length CRBN present in the tumor cells may be sufficient to confer drug sensitivity, especially for a more potent compound. Additionally, the putative variant protein product has not been observed in clinical samples due to the lack of a proper assay. Finally, the presence of *CRBN-Del-Exon10* in untreated patients raises additional questions about its “dominant negative” biological role, as CRBN itself is not essential in MM. Taken together, there are many important questions that need further investigation to address the biological basis for the function of the *CRBN-Del-Exon10* variant in terms of prognosis in general, or resistance to IMiD therapy in particular.

The significant number of patients with high *CRBN-Del-Exon10* variant in IMiD-treated RRMM patients potentially presents a targetable segment where the next generation CRBN-modulating agents (CELMoDs) such as iberdomide or CC-92480 may show clinical activity. However, current MM treatment strategies use combinations of IMiDs with proteasome inhibitors (PI) and steroids as the standard of care in NDMM and RRMM settings (Kumar et al., 2019). The significant increase in patients expressing the *CRBN-Del-Exon10* splice variant in RRMM opens the possibility to explore if there are ways to identify new therapeutics that could be targeted to these patients. Along with CELMoDs, drugs with new mechanisms of action are now also being developed in hematological malignancies. Among these are BCL2 inhibitors (e.g., venetoclax) and EZH2 inhibitors (e.g., tazemetostat), which have the potential to be also clinically relevant in MM (Tremblay-LeMay et al., 2018; Yap et al., 2019; Gupta et al., 2021). Indeed, venetoclax is in clinical development and has shown promise in patients harboring the t(11:14) translocation (Kumar et al., 2017). Recently, Gupta et al. (2021) reported preclinical analysis of MM cell lines and identified venetoclax sensitivity based on a gene expression signature, providing a biomarker-based approach for venetoclax. But so far, a rational combination of venetoclax with IMiDs or PI in subsets of biomarker-defined RRMM patients have not been proposed. Similarly, EZH2 inhibition was shown to be effective in MM cell lines, and clinical trials are currently exploring its potential in MM patients (Tremblay-LeMay et al., 2018; Yap et al., 2019), although not in a targeted segment.

Here, we analyzed genomic and transcriptomic profiles of *CRBN-Del-Exon10*–overexpressing NDMM and RRMM patient tumors and identified pro-inflammatory gene expression pathways to be highly associated with patients overexpressing the *CRBN-Del-Exon10* splice variant. In addition to IKZF1, a common substrate protein of the IMiD drugs, we identified two transcriptional master regulators, EZH2 and BATF, to be common across the data sets. We analyzed gene expression profiles in the samples and modeled their sensitivity to venetoclax or EZH2 inhibition by using gene expression–based sensitivity signatures (Gupta et al., 2021 and this work). Together, these analyses lead us to suggest the potential of a BCL2 inhibitor or EZH2 inhibitor as the targets for devising combination therapeutic approaches in this group of MM patients.

## Materials and Methods

Patient-level transcriptomic data and clinical information, including cytogenetics and progression/survival outcomes, were assessed. RNA extraction, library preparation, and sequencing for the Multiple Myeloma Research Foundation (MMRF), CC-4047-MM-010 and CC-220-MM-001, have been described previously (Walker et al., 2018; Gooding et al., 2021; Ortiz Estevez et al., 2021). NDMM patient data from the MMRF (N = 348) was used as discovery, and Toulouse [*N* = 127, all t(4:14) patients] as replication cohorts. RRMM patient data from clinical trials NCT01712789/CC-4047-MM-010 (*N* = 187) as discovery and NCT02773030/CC-220-MM-001 (*N* = 91) as replication cohorts were analyzed to investigate splicing and transcription of genes associated with high *CRBN-Del-Exon10* splice variant. The patient-level data included transcriptome, clinical demographics, and clinical outcomes. The NDMM and RRMM samples had sufficient purity (>85% tumor cells) for these analyses. The cytogenetics data were gathered from a previous study (Walker et al., 2018). The purity-adjusted cutoff for a high exon-10 spliced/full-length transcript ratio (2.6) was used to define exon-10 splice variant patients (Gooding et al., 2021). The limma–voom framework (R/Bioconductor) (Law et al., 2014) was used to identify differentially expressed genes among patients with exon-10 splice variant versus those with CRBN wild-type (WT) while controlling for covariates. The gene set variation analysis (GSVA) (Hanzelmann et al., 2013), as implemented in the GSVA package in R, was applied to explore the activated oncogenic signaling pathways; pathway signatures were obtained from KEGG (Kanehisa et al., 2017), HALLMARK (Liberzon et al., 2015), and REACTOME (Jassal et al., 2020) databases; and gene set enrichment analysis (GSEA) (Subramanian et al., 2005) was run to investigate functionally enriched processes in patients with high *CRBN-Del-Exon10* splice variant. Transcription factors and their regulons were obtained through the Encyclopedia of DNA Elements Project (ENCODE Project Consortium et al., 2020), and transcription factor enrichment analysis was performed by hypergeometric test to identify disease drivers in high *CRBN-Del-Exon10* expression patients.

## Results and Discussion

### High Expression of *CRBN-Del-Exon10* Splice Variant is Prognostic in Multiple Myeloma

Our previous report identified a subset of IMiD-resistant patients expressing high levels of *CRBN-Del-Exon10* (Gooding et al., 2021). Here, we explore its prevalence and co-occurrence in key MM subgroups (IgH translocation and hyperdiploidy) in NDMM and RRMM. We analyzed transcriptomic data from various MM data sets: for NDMM, a subset of the MMRF (discovery, *N* = 348) and Toulouse (replication, *N* = 127) and for RRMM, MM-010 (discovery, *N* = 172) and MM-001 (replication, *N* = 91). In aggregate, 8.3% of NDMM and 13.4% of RRMM patients expressed high levels of the variant, indicating its presence in untreated patients which increased in RRMM. Our previous analysis showed prognostic effects in high *CRBN-Del-Exon10* splice variant patients from a large RRMM data set (MM-010) and a large cohort of NDMM (MMRF) patients (Gooding et al., 2021). Here, we aimed to uncover the molecular characteristics of high *CRBN-Del-Exon10* splice variant patients using previous data sets and additional NDMM and RRMM cohorts.

### Myeloma Cells of Patients Expressing High Levels of *CRBN-Del-Exon10* Variant Show Upregulated Pro-Inflammatory Cytokine Pathways Which May Promote Myeloma Cell Growth

**FIGURE 1:**
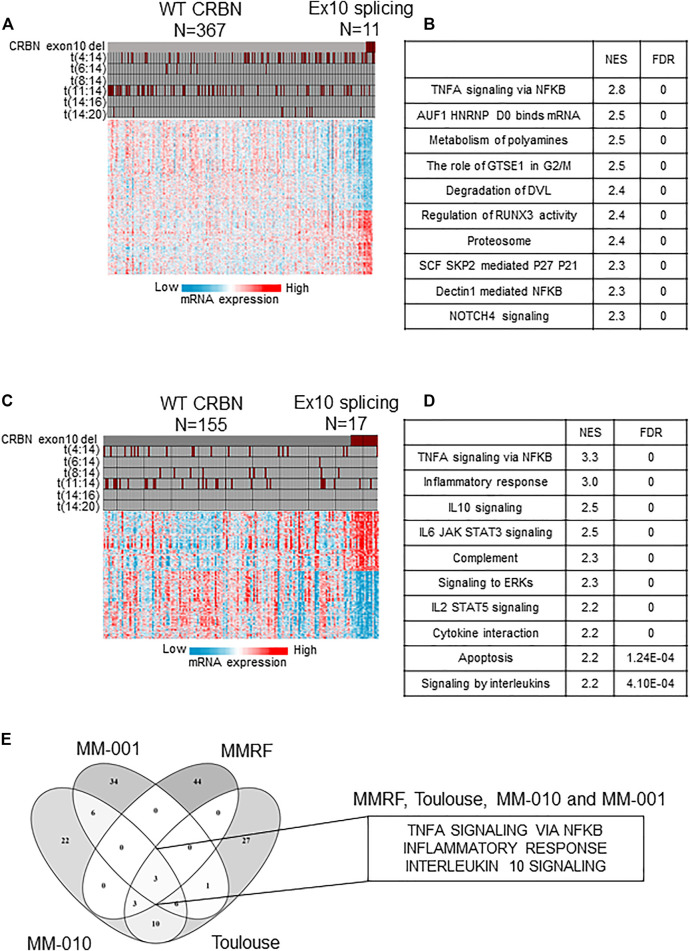
Transcriptomic characteristics of patient with high levels of *CRBN-Del-Exon10* splice variant and wild-type (WT) CRBN in newly diagnosed multiple myeloma (NDMM) and relapsed refractory multiple myeloma (RRMM) cohorts. **(A)** Gene expression profile with cytogenetic features of high levels of *CRBN-Del-Exon10* splice variant vs WT CRBN patients (MMRF cohort) and **(B)** functional enrichment of NDMM patients with *CRBN-Del-Exon10* splice variant vs WT CRBN patients (MMRF cohort). **(C)** Gene expression profile with cytogenetic features of *CRBN-Del-Exon10* splice variant vs WT CRBN patients (MM-010 cohort) and **(D)** functional enrichment of RRMM patients with high levels of *CRBN-Del-Exon10* splice variant vs WT CRBN patients (MM-010 cohort). **(E)** Comparison of functional enrichment analysis across NDMM and RRMM data sets (CRBN = cereblon, Ex10 = exon-10, NES = normalized enrichment score, FDR = false discovery rate).

We did not find evidence of significant association of a dysregulated splicing machinery (increased mutations in the splicing machinery or overall increase in alternative splicing) with high *CRBN-Del-Exon10* expression in the tumors of patients (Supplementary Tables S1–S4). We then analyzed transcriptome-wide data to identify differentially expressed pathways and genes between the high *CRBN-Del-Exon10*–expressing patients and the WT-expressing patients. GSEA showed that among the upregulated pathways, predominantly, the multiple immune system–related signaling pathways, including TNFA signaling via NFKB, inflammatory response, and IL1 and IL10 signaling, were significantly enriched in tumors expressing high levels of *CRBN-Del-Exon10* splice variant in NDMM (Figures 1A,B). These results are consistent with similar analyses performed on RRMM (MM-010 data set) in which TNFA signaling via NFKB, inflammatory response, IL10 signaling, and IL6/JAK/STAT3 signaling were also significantly enriched (Figures 1C,D). Finally, these observations have been further confirmed in two independent NDMM and RRMM patient cohorts (Toulouse and CC220-MM-001, respectively; Supplementary Figure S1A–B). A comparison of the top 50 enriched functional terms revealed three pathways (TNFA signaling via NFKB, inflammatory response, and IL10 signaling) consistently activated in high *CRBN-Del-Exon10*–expressing patients across all four data sets (Figure 1E) independent of the disease setting. These results show an unexpected correlation between high *CRBN-Del-Exon10* expression and upregulated pro-inflammatory immune pathway genes within the tumor cells. As this association is seen in untreated as well as in treated tumors, it suggests that the upregulated cytokine pathways may be intrinsically related to the specific splicing of CRBN rather than being linked to resistance to IMiD drugs *per se*. Finally, even though the samples are highly purified (typically >85% tumor cells), making it highly unlikely that the transcriptional data would be derived from contaminating immune cells (the traditional source of pro-inflammatory cytokines), our results do not preclude the possibility of the immune microenvironment inducing the expression of growth-promoting cytokines within the tumor before they are extracted for gene expression analysis. TME-dependent expression of immune genes in tumor cells has been demonstrated in solid tumors, where tumor-intrinsic inflammatory signals are known to result in an immunosuppressive TME and to provide a pro-growth milieu for tumor development (Elinav et al., 2013; Ragu et al., 2020; Saitoh and Oda, 2021).

### Master Regulators, BATF1 and EZH2, Are Key Transcription Factors Regulating Key Signaling Pathways in *CRBN-Del-Exon10*–Expressing Patients

**FIGURE 2:**
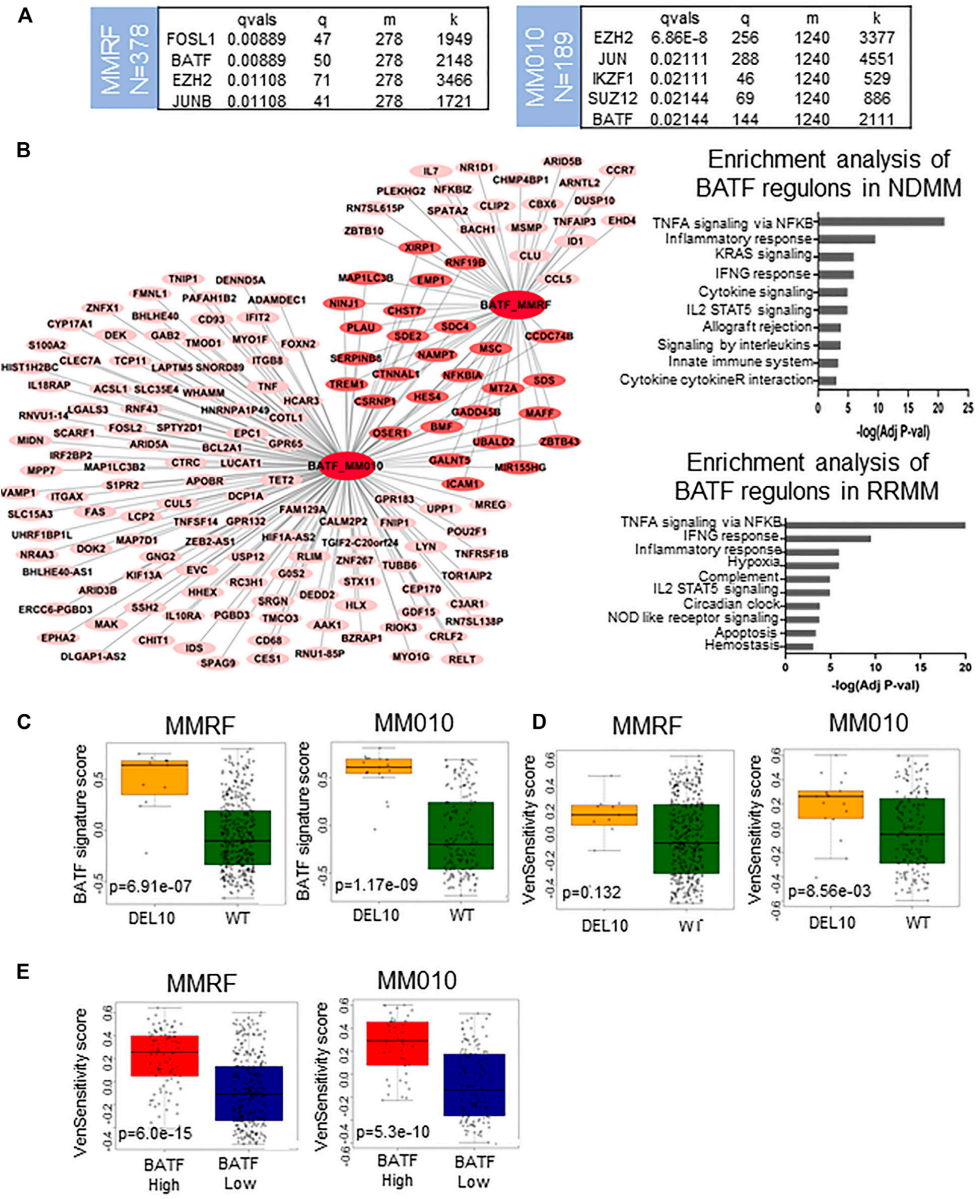
Transcription factor enrichment analysis of *CRBN-Del-Exon10* splice variant patients and their applications. **(A)** Enriched transcriptional factors that regulate activated genes in *CRBN-Del-Exon10* splice variant patients in newly diagnosed multiple myeloma (NDMM) and relapsed refractory multiple myeloma (RRMM) cohorts. **(B)** Transcriptional regulatory network of BATF including its upregulated direct targets and BATF-regulated oncogenic signaling pathways in MMRF and MM-010 data sets. **(C)** BATF signature activity in *CRBN-Del-Exon10* splice variant patients in MMRF and MM-010 data sets. **(D)** Venetoclax sensitivity activity score in *CRBN-Del-Exon10* splice variant patients compared to the wild type (WT) in MMRF and MM-010 data sets. **(E)** BATF signature score is associated with venetoclax sensitivity signature score.

We performed master regulator analyses to identify the key transcription factors that could regulate the signaling pathways in patients expressing high *CRBN-Del-Exon10* splice variant. BATF and EZH2 were identified as the only common transcription factors among NDMM (MMRF) and RRMM (MM-010). These results were confirmed in additional data sets (Figure 2A and Supplementary Figure S2A–C and Supplementary Figure S3A). As EZH2 inhibitors are in clinical development and BATF has been previously linked with sensitivity to venetoclax (Gupta et al., 2021), we focused on the potential of therapeutically targeting EZH2 or BATF regulatory networks in *CRBN-Del-Exon10*–expressing patients.

To identify the specific signaling pathways regulated by the two master regulators, we conducted functional enrichment analysis on their upregulated targets. The upregulated downstream effectors of BATF mainly played a role in TNFA signaling via NFKB, inflammatory and IFNG responses, and IL2/STAT5 signaling pathways in both NDMM and RRMM cohorts (Figure 2B). Similarly, the upregulated downstream effectors of EZH2 mainly played a role in TNFA signaling via NFKB in NDMM and RRMM (Supplementary Figure S3B).

We next defined specific activity signatures for BATF and EZH2 regulons by including common upregulated target genes of each transcription factor across data sets. BATF and EZH2 signatures included 36 and 54 upregulated direct target genes, respectively, and only nine genes were common among these signatures (including BMF, CSRNP1, HES4, ICAM1, MIR155HG, MSC, MT2A, PLAU, and UBALD2). BATF signature activity was significantly higher in high *CRBN-Del-Exon10*–expressing patients than in WT-expressing patients in NDMM (MMRF) (*p* = 6.91E-07) and RRMM (MM-010) (*p* = 1.17E-07) (Figure 2C). This was confirmed in the independent data sets for NDMM (Toulouse) (*p* = 2.0E-07) and RRMM (MM-001) (*p* = 2.58E-04) patients (Supplementary Figure S2A). EZH2 signature activity was similarly significantly higher in high *CRBN-Del-Exon10* splice variant patients across data sets (Supplementary Figure S3C). This analysis prompted us to consider the effects of EZH2 or BATF inhibitors in this subset of patients.

### BATF- and EZH2-Regulated Cytokine Pathways Could Be Targeted by Venetoclax and/or EZH2 Inhibitors

**FIGURE 3:**
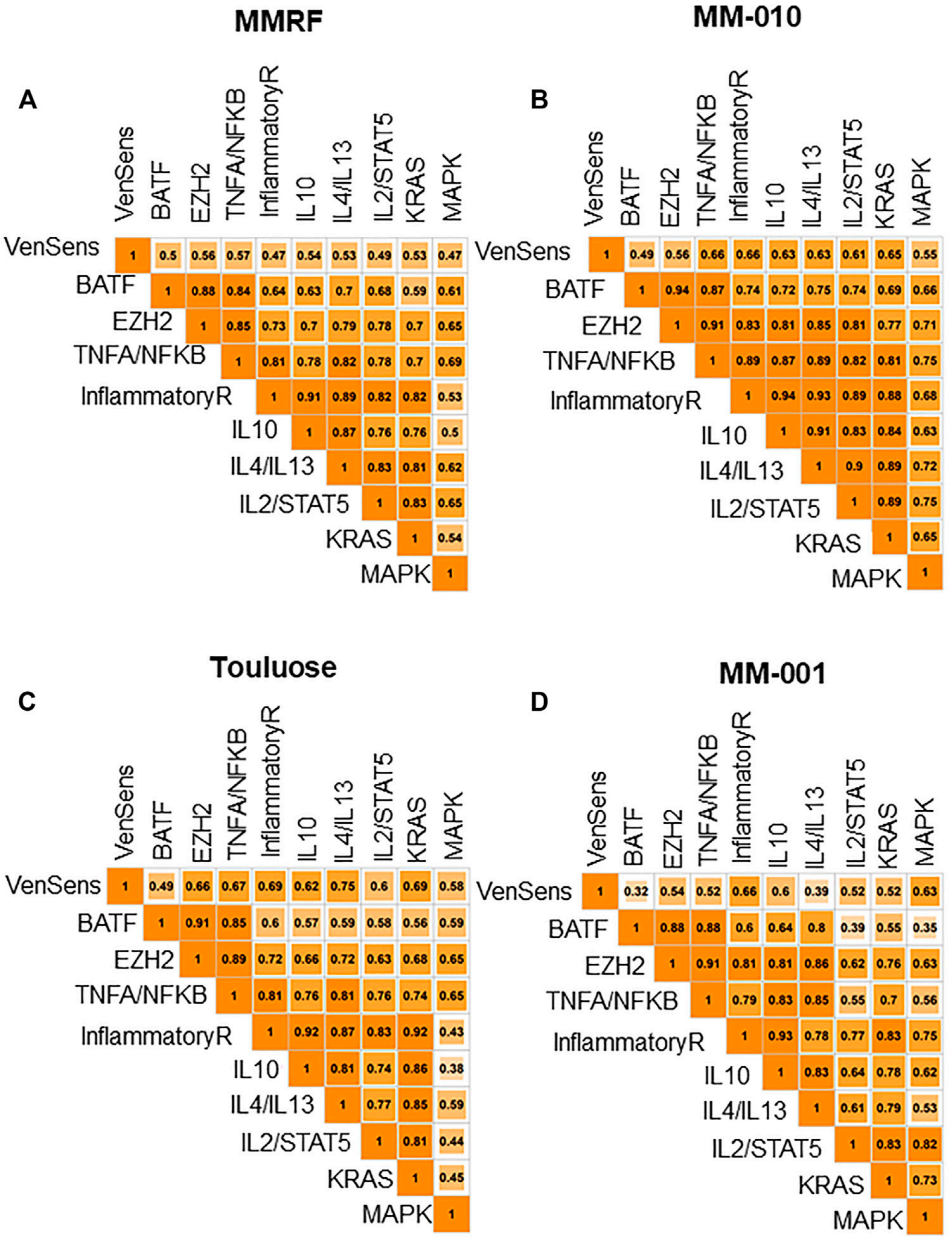
**(A,B)** Venetoclax sensitivity signature score is associated with BATF-dependent transcriptional programs and TNFA signaling via NFKB in MMRF and MM-010. **(C**,**D)** Venetoclax sensitivity signature score is associated with BATF-dependent transcriptional programs and TNFA signaling via NFKB in Toulouse and MM-001.

Several ongoing clinical trials are testing EZH2 inhibitors in MM (Yap et al., 2019), and preclinical studies have also provided a rationale for the therapeutic relevance of EZH2 inhibitors in MM (Pawlyn et al., 2017; Tremblay-LeMay et al., 2018). Although there is no known BATF inhibitor in the clinic, a BATF-mediated transcriptional program was shown to correlate with venetoclax sensitivity in MM cell lines (Gupta et al., 2021). Gupta *et al.* identified 110 genes that were upregulated in cells from venetoclax-sensitive patients, and we applied this set of genes as a venetoclax sensitivity signature, calculating a venetoclax sensitivity score for each patient in the NDMM and RRMM cohorts. Then, we compared the activity of BATF (based on the signature described in the previous section), venetoclax sensitivity, and the oncogenic signaling pathways in our data sets. Our analyses showed that high *CRBN-Del-Exon10*–expressing patients, as well as patients with high BATF activity, had higher venetoclax sensitivity signature scores (Figures 2D,E). Furthermore, we found that the BATF-dependent transcriptional program was significantly associated with not only venetoclax sensitivity but also TNFA signaling via NFKB pathway activation in all four data sets (MM-010: R = 0.87; MM-001: R = 0.88; MMRF: R = 0.84; Toulouse: R = 0.85, *p* = 4.44e-16) (Figures 3A–D). Additionally, the EZH2 signature score was identified as highly correlated with not only TNFA signaling via NFKB pathway activation in NDMM (MMRF, R = 0.85; Toulouse, R = 0.89) and RRMM (MM-010, R = 0.91; MM-001, R = 0.91) but also BATF signature scores in NDMM (MMRF, R = 0.88; Toulouse, R = 0.91) and RRMM (MM-010, R = 0.94; MM-001, R = 0.88), and venetoclax sensitivity signature score in NDMM (MMRF, R = 0.56; Toulouse, R = 0.66) and RRMM (MM-010: R = 0.56, *p* = 8.88e-16; MM-001: R = 0.54, *p* = 1.49e-7). All correlations were significantly different from zero, and all had *p*-values less than 2.2e-16 except where otherwise noted. These results suggest that both venetoclax and an EZH2 inhibitor may be clinically active in patients expressing high *CRBN-Del-Exon10.*

In summary, high *CRBN-Del-Exon10* variant expression identifies a MM patient group that harbors diverse genetic features, but a common transcriptional program with pro-growth immune signaling regardless of the clinical setting. While the biological connections between high *CRBN-Del-Exon10* expression, function, and immune signaling pathways are unknown, these results suggest its potential link with both MM cell growth and IMiD resistance. Based on the understanding of tumor-intrinsic inflammatory signaling pathways in solid tumors and the data presented in this article, it is possible that chromosomal instability and DNA damage response/replication stress may be linked to the upregulation of pro-inflammatory cytokines (e.g., TNFA, IL6) and immunosuppressive TME signaling (IL10, IL4, IL13) in patients expressing high *CRBN-Del-Exon10* splice variant (Elinav et al., 2013; Ragu et al., 2020; Saitoh and Oda, 2021).

Our analysis identified two potentially actionable transcription factors, BATF and EZH2, that can be targeted by venetoclax (a BCL2 inhibitor) and tazemetostat (EZH2 inhibitor), respectively. Although EZH2 inhibitors have not been tested in BATF high tumors (or cell line models), based on our analysis, it is possible that venetoclax-sensitive cells could be also sensitive to EZH2 inhibitors. Combination of these agents with new Cereblon-targeting agents (CELMoDs), which might overcome the resistance conferred by the expression of high *CRBN-Del-Exon10* splice variant in IMiD-resistant/refractory patients, would be an attractive possibility to test in the clinic as a novel combination therapy for this molecularly defined subset of MM patients.

We also point out (Thakurta et al., 2021) some limitations of our analyses that require additional work. First, while we showed a clear association between the transcriptional programs and the expression of high *CRBN-Del-Exon10* splice variant in patient tumor cells, identification of a clear biological connection between them was outside the scope of this investigation. Exploration of the association of *CRBN-Del-Exon10* with known genomic biomarkers, patient subsets, and disease settings did not provide any clear guidance to understand the biological connection between *CRBN-Del-Exon10* splice variant and immune cytokine expression. Secondly, our working definition of high *CRBN-Del-Exon10* splice variant was variant/full length ratio ≥2.6 based on our published work (Gooding et al., 2021). Whether a different and more optimized value would be better suited to define the patients with high *CRBN-Del-Exon10* splice variant needs analyses of more patient data. Finally, our proposal for combining the novel agents will require some preclinical experimental validation of the proposed combinations. This will require establishing proper preclinical model systems (that express high *CRBN-Del-Exon10)* to test drug sensitivities (Ito et al., 2010; Thakurta et al., 2021).

## Data Availability

Disclosure of patient level data requires review of requests consistent with patient consent confidentiality and ethical standards. Please contact the corresponding authors for data access requests.

## Supplementary Material

Supplementary Figure S1 Transcriptomic characteristics of patients with high levels of *CRBN-Del-Exon10* splice variant in relapsed refractory multiple myeloma (RRMM) and newly diagnosed multiple myeloma (NDMM) cohorts as confirmation. **(A)** Gene expression profile with cytogenetic features of high levels of *CRBN-Del-Exon10* splice variant vs wild type (WT) CRBN patients (Toulouse cohort), **(B)** Functional enrichment of NDMM patients with *CRBN-Del-Exon10* splice variant vs WT CRBN patients (Toulouse cohort). **(C)** Gene expression profile with cytogenetic features of *CRBN-Del-Exon10* splice variant vs WT CRBN patients (MM-001 cohort) and **(D)** Functional enrichment of RRMM patients with *CRBN-Del-Exon10* splice variant vs WT CRBN patients (MM-001 cohort).

Supplementary Figure S2 BATF signature activity enriched in *CRBN-Del-Exon10* splice variant patients in conformation data sets, Toulouse and MM-001 data sets. **(A)** BATF signature activity in *CRBN-Del-Exon10* splice variant patients **(B)** Venetoclax sensitivity activity score in *CRBN-Del-Exon10* splice variant patients compared to wild type (WT) CRBN. **(C)** BATF signature score is associated with venetoclax sensitivity signature score.

Supplementary Figure S3 Transcription factor enrichment analysis of *CRBN-Del-Exon10* splice variant patients and their applications in confirmation data sets. **(A)** Enriched transcriptional factors that regulates activated genes in *CRBN-Del-Exon10* splice variant patients in additional NDMM and RRMM cohorts. **(B)** Transcriptional regulatory network of EZH2 including its up-regulated direct targets and EZH2 regulated oncogenic signaling pathways in MMRF and MM-010 data sets. **(C)** EZH2 signature activity in *CRBN-Del-Exon10* splice variant patients in NDMM and RRMM data sets.

## References

[B1] Chamberlain P. P. Lopez-Girona A. Miller K. Carmel G. Pagarigan B. Chie-Leon B. (2014). Structure of the Human Cereblon-DDB1-Lenalidomide Complex Reveals Basis for Responsiveness to Thalidomide Analogs. Nat. Struct. Mol. Biol. 21 (9), 803–809. 10.1038/nsmb.2874 25108355

[B2] Elinav E. Nowarski R. Thaiss C. A. Hu B. Jin C. Flavell R. A. (2013). Inflammation-induced Cancer: Crosstalk between Tumours, Immune Cells and Microorganisms. Nat. Rev. Cancer 13, 759–771. 10.1038/nrc3611 24154716

[B17] ENCODE Project Consortium, Moore J. E. Purcaro M. J. Pratt H. E. Epstein C. B. Shoresh N. Adrian J. (2020). Expanded Encyclopaedias of DNA Elements in the Human and Mouse Genomes. Nature 583 (7818), 699–710. 10.1038/s41586-020-2493-4 32728249 PMC7410828

[B3] Franssen L. E. Nijhof I. S. Couto S. Levin M.-D. Bos G. M. J. Broijl A. (2018). Cereblon Loss and Up-Regulation of C-Myc Are Associated with Lenalidomide Resistance in Multiple Myeloma Patients. Haematologica 103, e368–e371. 10.3324/haematol.2017.186601 29545338 PMC6068039

[B4] Gandhi A. K. Mendy D. Waldman M. Chen G. Rychak E. Miller K. (2014). Measuring Cereblon as a Biomarker of Response or Resistance to Lenalidomide and Pomalidomide Requires Use of Standardized Reagents and Understanding of Gene Complexity. Br. J. Haematol. 164 (2), 233–244. 10.1111/bjh.12622 24206017 PMC4253085

[B5] Gooding S. Ansari-Pour N. Towfic F. Ortiz Estévez M. Chamberlain P. P. Tsai K.-T. (2021). Multiple Cereblon Genetic Changes Are Associated with Acquired Resistance to Lenalidomide or Pomalidomide in Multiple Myeloma. Blood 137 (2), 232–237. 10.1182/blood.2020007081 33443552 PMC7893409

[B6] Gupta V. A. Barwick B. G. Matulis S. M. Shirasaki R. Jaye D. L. Keats J. J. (2021). Venetoclax Sensitivity in Multiple Myeloma Is Associated with B-Cell Gene Expression. Blood 137 (26), 3604–3615. 10.1182/blood.2020007899 33649772 PMC8462405

[B7] Hänzelmann S. Castelo R. Guinney J. (2013). GSVA: Gene Set Variation Analysis for Microarray and RNA-Seq Data. BMC Bioinformatics 14, 7. 10.1186/1471-2105-14-7 23323831 PMC3618321

[B8] Ito T. Ando H. Suzuki T. Ogura T. Hotta K. Imamura Y. (2010). Identification of a Primary Target of Thalidomide Teratogenicity. Science 327, 1345–1350. 10.1126/science.1177319 20223979

[B9] Jassal B. Matthews L. Viteri G. Gong C. Lorente P. Fabregat A. (2020). The Reactome Pathway Knowledgebase. Nucleic Acids Res. 48 (D1), D498–D503. 10.1093/nar/gkz1031 31691815 PMC7145712

[B10] Kanehisa M. Furumichi M. Tanabe M. Sato Y. Morishima K. (2017). KEGG: New Perspectives on Genomes, Pathways, Diseases and Drugs. Nucleic Acids Res. 45 (D1), D353–D361. 10.1093/nar/gkw1092 27899662 PMC5210567

[B11] Kortüm K. M. Mai E. K. Hanafiah N. H. Shi C.-X. Zhu Y.-X. Bruins L. (2016). Targeted Sequencing of Refractory Myeloma Reveals a High Incidence of Mutations in CRBN and Ras Pathway Genes. Blood 128, 1226–1233. 10.1182/blood-2016-02-698092 27458004 PMC5524534

[B12] Kumar S. Kaufman J. L. Gasparetto C. Mikhael J. Vij R. Pegourie B. (2017). Efficacy of Venetoclax as Targeted Therapy for Relapsed/refractory T(11;14) Multiple Myeloma. Blood 130 (22), 2401–2409. 10.1182/blood-2017-06-788786 29018077

[B13] Kumar S. K. Callander N. S. Hillengass J. Liedtke M. Baljevic M. Campagnaro E. (2019). NCCN Guidelines Insights: Multiple Myeloma, Version 1.2020. J. Natl. Compr. Canc Netw. 17 (10), 1154–1165. 10.6004/jnccn.2019.0049 31590151

[B14] Law C. W. Chen Y. Shi W. Smyth G. K. (2014). Voom: Precision Weights Unlock Linear Model Analysis Tools for RNA-Seq Read Counts. Genome Biol. 15 (2), R29. 10.1186/gb-2014-15-2-r29 24485249 PMC4053721

[B15] Liberzon A. Birger C. Thorvaldsdóttir H. Ghandi M. Mesirov J. P. Tamayo P. (2015). The Molecular Signatures Database Hallmark Gene Set Collection. Cel Syst. 1 (6), 417–425. 10.1016/j.cels.2015.12.004 PMC4707969 26771021

[B16] Lopez-Girona A. Mendy D. Ito T. Miller K. Gandhi A. K. Kang J. (2012). Cereblon Is a Direct Protein Target for Immunomodulatory and Antiproliferative Activities of Lenalidomide and Pomalidomide. Leukemia 26 (11), 2326–2335. 10.1038/leu.2012.119 22552008 PMC3496085

[B18] Ortiz-Estévez M. Towfic F. Flynt E. Stong N. Jang I. S. Wang K. (2021). Integrative Multi-Omics Identifies High Risk Multiple Myeloma Subgroup Associated with Significant DNA Loss and Dysregulated DNA Repair and Cell Cycle Pathways. BMC Med. Genomics 14 (1), 295. 10.1186/s12920-021-01140-5 34922559 PMC8684160

[B19] Pawlyn C. Bright M. D. Buros A. F. Stein C. K. Walters Z. Aronson L. I. (2017). Overexpression of EZH2 in Multiple Myeloma Is Associated with Poor Prognosis and Dysregulation of Cell Cycle Control. Blood Cancer J. 7 (3), e549. 10.1038/bcj.2017.27 28362441 PMC5380911

[B20] Ragu S. Matos-Rodrigues G. Lopez B. S. (2020). Replication Stress, DNA Damage, Inflammatory Cytokines and Innate Immune Response. Genes 11 (4), 409. 10.3390/genes11040409 PMC7230342 32283785

[B21] Saitoh T. Oda T. (2021). DNA Damage Response in Multiple Myeloma: The Role of the Tumor Microenvironment. Cancers 13 (3), 504. 10.3390/cancers13030504 33525741 PMC7865954

[B22] Subramanian A. Tamayo P. Mootha V. K. Mukherjee S. Ebert B. L. Gillette M. A. (2005). Gene Set Enrichment Analysis: a Knowledge-Based Approach for Interpreting Genome-wide Expression Profiles. Proc. Natl. Acad. Sci. 102 (43), 15545–15550. 10.1073/pnas.0506580102 16199517 PMC1239896

[B23] Thakurta A. Gandhi A. K. Waldman M. F. Bjorklund C. Ning Y. Mendy D. (2014). Absence of Mutations in Cereblon (CRBN) and DNA Damage-Binding Protein 1 (DDB1) Genes and Significance for IMiD Therapy. Leukemia 28 (5), 1129–1131. 10.1038/leu.2013.315 24166296 PMC4017253

[B24] Thakurta A. Pierceall W. E. Amatangelo M. D. Flynt E. Agarwal A. (2021). Developing Next Generation Immunomodulatory Drugs and Their Combinations in Multiple Myeloma. Oncotarget 12 (15), 1555–1563. 10.18632/oncotarget.27973 34316334 PMC8310669

[B25] Tremblay-LeMay R. Rastgoo N. Pourabdollah M. Chang H. (2018). EZH2 as a Therapeutic Target for Multiple Myeloma and Other Haematological Malignancies. Biomark Res. 6, 34. 10.1186/s40364-018-0148-5 30555699 PMC6286605

[B26] Walker B. A. Mavrommatis K. Wardell C. P. Ashby T. C. Bauer M. Davies F. E. (2018). Identification of Novel Mutational Drivers Reveals Oncogene Dependencies in Multiple Myeloma. Blood 132 (6), 587–597. 10.1182/blood-2018-03-840132 29884741 PMC6097138

[B27] Yap T. A. Winter J. N. Giulino-Roth L. Longley J. Lopez J. Michot J.-M. (2019). Phase I Study of the Novel Enhancer of Zeste Homolog 2 (EZH2) Inhibitor GSK2816126 in Patients with Advanced Hematologic and Solid Tumors. Clin. Cancer Res. 25 (24), 7331–7339. 10.1158/1078-0432.18-4121 31471312 PMC7377921

