# Supplementary figures and images for: Multiple Myeloma Patient Tumors With High Levels of Cereblon Exon-10 Deletion Splice Variant Upregulate Clinically Targetable Pro-Inflammatory Cytokine Pathways
Source: Front Genet. 2022 Feb 9;13:831779. doi: 10.3389/fgene.2022.831779 (PMC8864318; doi:10.3389/fgene.2022.831779)

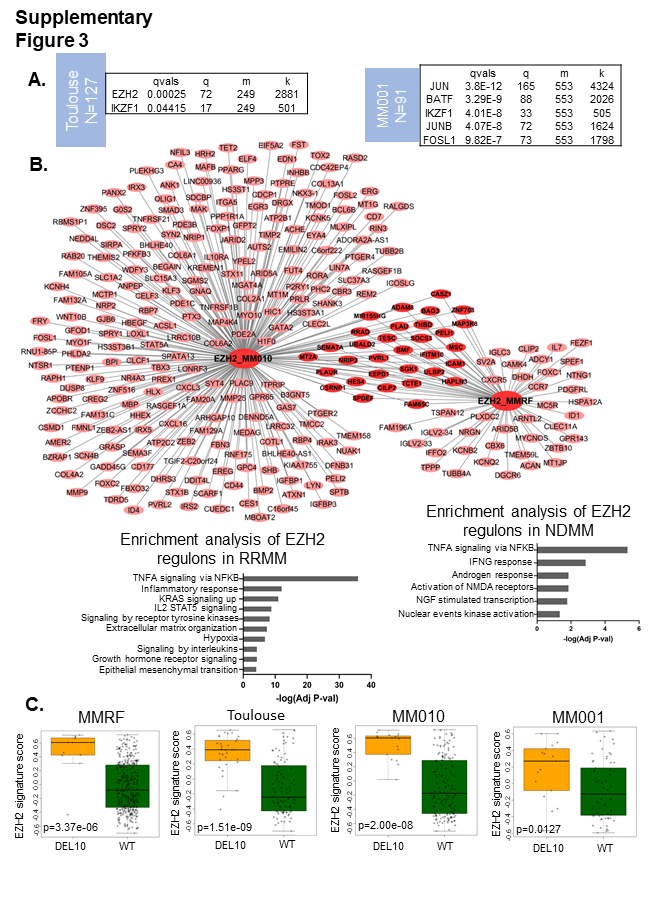

Supplement: Supplementary file 1 [file Image3.tif]

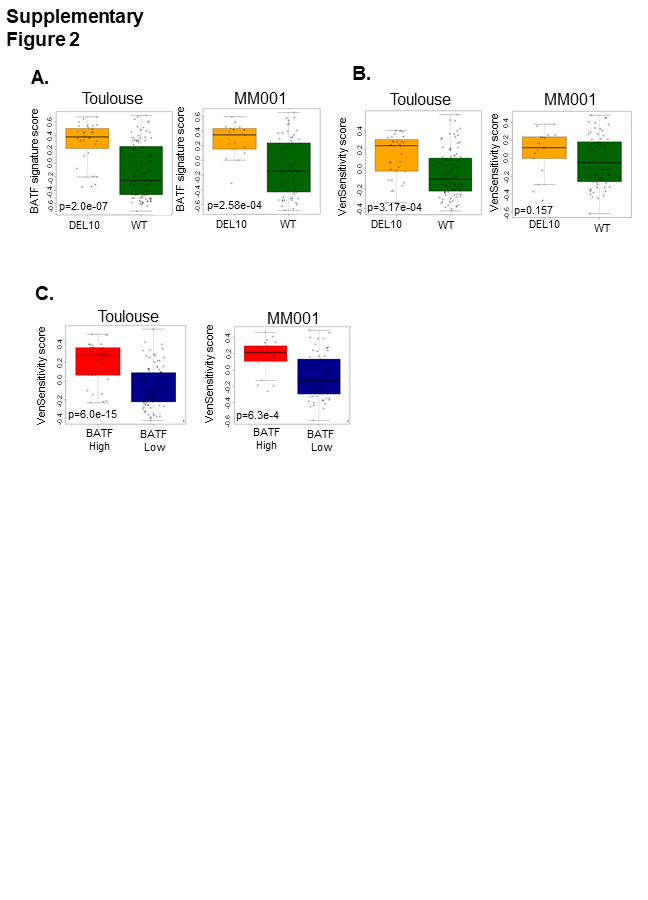

Supplement: Supplementary file 2 [file Image2.tif]

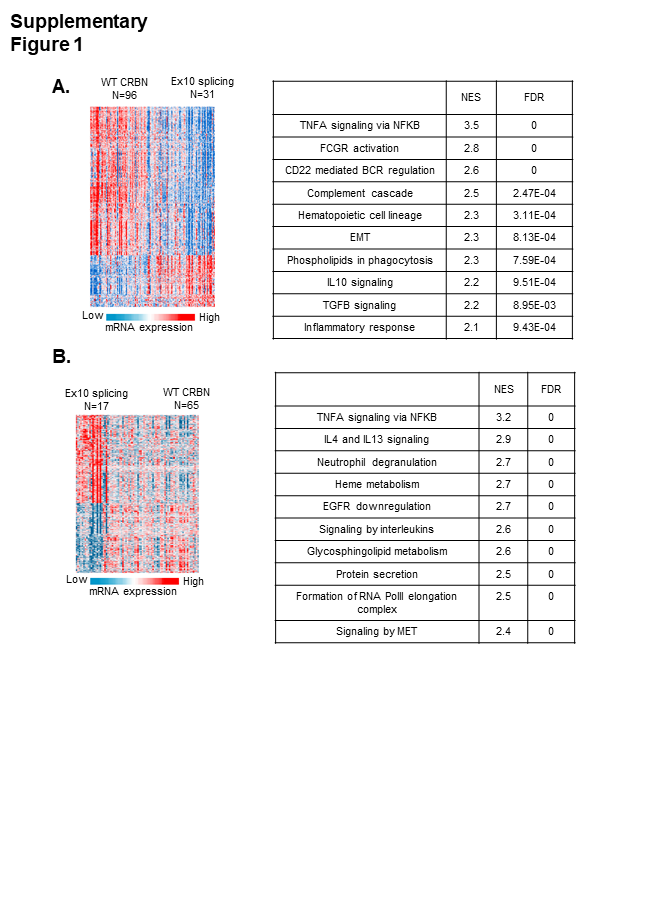

Supplement: Supplementary file 3 [file Image1.tif]
